# Updated Management Guidelines for *Clostridioides difficile* in Paediatrics

**DOI:** 10.3390/pathogens9040291

**Published:** 2020-04-16

**Authors:** Margherita Gnocchi, Martina Gagliardi, Pierpacifico Gismondi, Federica Gaiani, Gian Luigi de’ Angelis, Susanna Esposito

**Affiliations:** 1 Pediatric Clinic, Pietro Barilla Children’s Hospital, Department of Medicine and Surgery, University of Parma, 43126 Parma, Italy; margherita.gnocchi@gmail.com (M.G.); marty.gagliardi@hotmail.it (M.G.); pgismondi@ao.pr.it (P.G.); 2 Unit of Gastroenterology and Digestive Endoscopy, Department of Medicine and Surgery, University of Parma, 43126 Parma, Italy; federica.gaiani@unipr.it (F.G.); gianluigi.deangelis@unipr.it (G.L.d.A.); 3 Pietro Barilla Children’s Hospital, Department of Medicine and Surgery, University of Parma, Via Gramsci 14, 43126 Parma, Italy

**Keywords:** *Clostridioides difficile*, *Clostridium difficile* infection, faecal microbiota transplant, fidaxomicin, vancomycin

## Abstract

*Clostridioides difficile*, formerly known as *Clostridium difficile,* causes infections (CDI) varying from self-limited diarrhoea to severe conditions, including toxic megacolon and bowel perforation. For this reason, a prompt diagnosis is fundamental to early treatment and the prevention of transmission. The aim of this article is to review diagnostic laboratory methods that are now available to detect *C. difficile* and to discuss the most recent recommendations on CDI treatment in children. Currently, there is no consensus on the best method for detecting *C. difficile*. Indeed, none of the available diagnostics possess at the same time high sensitivity and specificity, low cost and rapid turnaround times. Appropriate therapy is targeted according to age, severity and recurrence of the episode of infection, and the recent availability of new antibiotics opens new opportunities. De-escalation of antibiotics that are directly associated with CDI remains a priority and the cautious use of probiotics is recommended. Vancomycin represents the first-line therapy for CDI, although in children metronidazole can still be used as a first-line drug. Fidaxomicin is a new treatment option with equivalent initial response rates as vancomycin but lower relapse rates of CDI. Faecal microbiota transplantation should be considered for patients with multiple recurrences of CDI. Monoclonal antibodies and vaccines seem to represent a future perspective against CDI. However, only further studies will permit us to understand whether these new approaches could be effective in therapy and prevention of CDI in paediatric populations.

## 1. Introduction

*Clostridioides difficile*, formerly known as *Clostridium difficile* before reclassification occurred in 2016 [1], is a spore-forming, anaerobic, Gram-positive bacterium [2]. It is ubiquitous in nature, especially in soil, and can be transmitted from human-to-human through the faecal–oral route or by direct exposure to the contaminated environment. *C. difficile* is considered the major cause of healthcare-associated diarrhoea, and even though this pathogen is more common in adults, in recent years, its rate has also increased in the paediatric population, both in hospital settings and in the community [3,4,5]. *C. difficile* colonizes the large intestine and produces two different proteins (toxins A and B) that are responsible for clinical disease. In particular, risk factors that alter the composition or barrier functions of the gut microbiota allow *C. difficile* to spread in the large intestine, and cause different degrees of colitis [6]. *C. difficile* infection (CDI) may therefore vary from self-limited diarrhoea to severe conditions, such as toxic megacolon and bowel perforation [3,6].

*C. difficile* colonization is far more frequent in the paediatric population than in adults, and this is the reason why most infants with evidence of *C. difficile* in laboratory testing are asymptomatic [3]. This may be explained by the absence of toxin-binding receptors in children’s immature intestinal mucosa, as seen in animal models [7]. *C. difficile* colonization in children varies widely, with incidence percentages being higher in neonates and in the first months of age. The *C. difficile* carriage rate in neonates ranges between 25% and 30%, then it lowers to 10–25% in infants from 1 to 12 months and to 5–10% in children over 1 year of age, while by 3 years, the prevalence is similar to that observed in adults (0–3%). Interestingly, excluding the neonatal population, comparable percentages of *C. difficile* colonization were observed in hospitalized infants and healthy age-matched outpatients [8].

Symptoms are rarely reported before 24 months of age, even though asymptomatic colonization may represent a source of transmission of the bacillus to others [9]. Both clinical illness and colonization are related to specific risk factors [10]. Asymptomatic colonization may be promoted by long hospitalization in neonatal units, early and multiple antibiotic administration and environmental exposure, while breast feeding, the absence of toxin-specific receptors in babies’ immature gut mucosa, fewer pathogenetic *C. difficile* strains and the production of specific antibodies against *C. difficile* toxins all represent protective factors [11]. The development of *C. difficile* clinical illness in children is the result of an altered balance between the host and the bacterium due to multiple factors. Recent antibiotic exposure, and particularly use of multiple antibiotics, is considered the most important risk factor for CDI because of the modifications of the normal intestinal flora [12].

Moreover, gastric acid suppression (i.e., use of proton-pump inhibitors or histamine-2-receptor antagonists) may promote *C. difficile* colonization of the large intestine, as can prolonged nasogastric tube insertion, gastrointestinal surgery, repeated enemas, gastrostomy and jejunostomy tubes and other medications including immunosuppressive drugs [10,13]. On the other hand, recognized host risk factors such as significant underlying chronic disease, immunosuppressive conditions, cancer, solid organ transplantation, renal insufficiency, cystic fibrosis and inflammatory bowel disease can contribute to CDI development [10,14,15].

Furthermore, another important factor to consider in the pathogenesis of *C. difficile* clinical illness is microbe virulence. The emergence of epidemic toxin-producing *C. difficile* strains, such as North American pulsed field type 1 (NAP1) or ribotype 027, is observed in more severe disease and the ability to infect children with neither a history of hospitalization nor recent use of antibiotics [16,17]. These strains are endemic in the US, Canada and Europe and may have a role in CDI epidemiology in children [4].

Clinical manifestations of CDI can be extremely different and vary from watery or bloody diarrhoea to toxic megacolon. Most children with a symptomatic infection are presented with a fever, mild to moderate diarrhoea, abdominal pain, anorexia and, in more severe cases, pseudomembranous colitis on endoscopy or histopathology, pneumatosis intestinalis, intestinal perforation or toxic megacolon [18]. For this reason, a prompt diagnosis is fundamental to early treatment and the prevention of transmission.

Currently, many different laboratory methods can be used to detect *C. difficile* in both paediatric and adult populations, even though there is not yet full accordance on what should be the best algorithm for diagnosing CDI. In addition, two guidelines for *C. difficile* have been proposed, but few data are available on their efficacy and safety in paediatric age groups. The aim of this article is to review diagnostic laboratory methods that are now available to detect *C. difficile* and to discuss the most recent recommendations on CDI therapy in children. The references of this review were identified through PubMed. We collected articles from the last ten years of literature (2010–2020) searching for “*Clostridium difficile*”, “*Clostridioides*”, “*Clostridium* therapy”, “*Clostridium* paediatric infection”, “*Clostridium* diagnosis in children”, “*Clostridium* therapy”, “fidaxomicin”, “fidaxomicin in children”, and “faecal microbiota transplant”.

## 2. Diagnosis

The diagnosis of CDI is performed considering both symptoms (diarrhoea) and laboratory tests. For this reason, it is recommended to perform *C. difficile* laboratory testing only in symptomatic children affected by prolonged or worsening diarrhoea, defined as a decrease in the consistency of stools associated with an increase in the frequency of evacuations (more than 3 in a 24-h period) or by more threatening symptoms such as bloody diarrhoea [19]. 

Due to the high prevalence of asymptomatic colonization in younger children, the American Academy of Paediatrics (AAP) and the Infectious Diseases Society of America (IDSA) recommend avoiding routine testing for CDI in neonates and infants (<1 year of life) with diarrhoea; instead, *C. difficile* should be considered a possible aetiology of clinical disease in children 1–3 years old, even though alternative aetiologies (in particular viral) must be ruled out first [4,19]. Indeed, approximately 20% of young patients in whose stool *C. difficile* has been found to have at the same time a positivity for other pathogens [20,21]. Therefore, under the age of 3 years, other diagnostic tests are mandatory to explain the cause of diarrhoea.

Moreover, as recommended by the IDSA recent update, the presence of *C. difficile* has to be investigated in children older than 2 years with diarrhoea and specific risk factors (i.e., recent multiple antimicrobial therapy, immunosuppression, inflammatory bowel disease (IBD) and Hirschsprung disease) because of their greater susceptibility or history of exposure to healthcare facilities [21]. The diagnostic approach to CDI in children older than 3 years is the same as that recommended for the adult population, although also in this age group as in younger children testing for more likely cause of diarrhoea (i.e., gastrointestinal virus detection) is recommended [19].

Many diagnostic methods with different targets can be used to detect *C. difficile* in stool specimens. Most techniques aim to detect pathogenic C. *difficile* toxins, such as the cell culture cytotoxicity neutralization assay (CCNA) and enzyme immunoassays (EIAs). EIAs also exist for another abundant *C. difficile* antigen (glutamate dehydrogenase), which are sensitive but not specific, as this antigen can be found in non-pathogenic, non-toxigenic strains of *C. difficile*. Other available diagnostics include a culture of *C. difficile* toxigenic strains and nucleic acid amplification tests. The recent introduction in many laboratories of the latter molecular technologies, targeting mostly toxin genes has improved the sensitivity and speed of *C. difficile* diagnosis [22], but raising issues of specificity for clinical disease vs. colonization.

Regardless of the diagnostics used, adequate storage conditions are extremely important to prevent sample degradation that may result in false negative tests. Once the stool specimen is collected, it has to be sent to the laboratory and processed within two hours [23]; if this is not possible, stools should be stored at 4 °C for a maximum of 72 h [23]. Moreover, any specific antimicrobial therapy should be administered after collecting stool samples to avoid false negative results. Sunkesula et al. showed that the initiation of empiric treatment in patients affected by CDI led to increasing percentages of conversion from positive to negative results after some days of therapy [24,25]. Therefore, if antibiotics are administered before collecting patients’ stool samples, a negative test result should not exclude CDI.

CCNA has been used for many years to directly detect *C. difficile*-free toxins in patients’ stool and is characterized by both high sensitivity and specificity. For this reason, it used to be the reference method in the past but has now been abandoned by most laboratories because of its slow turnaround time (the cytopathic effect is evaluated at 24 and 48 h) and lack of standardization due to the overall complexity of this procedure [24].

Toxigenic culture (TC) remains one of the reference procedures to detect *C. difficile*. It first allows the isolation of the bacillus on a selective medium and then demonstrates *C. difficile*’s ability to produce toxins in vitro. Although this method is characterized by a long turnaround time as with CCNA, it is necessary to identify isolates to test antimicrobial susceptibility and *C. difficile* strain typing [25].

One of the most common tests used today is EIA for detecting different *C. difficile* products, such as toxin A and B or glutamate dehydrogenase (GDH), a highly conserved metabolic enzyme expressed by all strains of *C. difficile*, both toxigenic and non-toxigenic. Many commercial EIAs are now available and provide rapid results with a relative ease of use [4,19]. Given the overall poor sensitivity and specificity of toxin EIAs and the low specificity of GDH immunoassays, these two methods are now usually combined in diagnostic algorithms to optimize *C. difficile* detection. GDH EIAs have also been proposed by different guidelines as screening tests, noting that positive results always need to be confirmed by more specific methods that are able to detect *C. difficile* toxins, considering that these latter are responsible for clinical illness.

Among molecular technologies, nucleic acid amplification tests (NAATs) based on real-time PCR, loop-mediated isothermal amplification or microarray technologies are able to identify different target genes, including those encoding toxins (*tcdA* and *tcdB*) or 16S ribosomal RNA (not available as commercial diagnostic kits) and have an overall greater sensitivity than toxin EIAs and comparable turnaround times [26]. Concerns have been raised about these assays detecting carriers of *C. difficile* with alternative aetiologies of their diarrhoea [27] 

Currently, there is no consensus on what is the best method for detecting *C. difficile*. The European Society of Clinical Microbiology and Infectious Diseases (ESCMID) recommends a multistep algorithm based on a first screening step with NAATs or GDH immunoassay (highly sensitive methods) followed, in the case of positivity, by a confirmatory test (CCNA) to directly detect toxins in stool specimens. Alternatively, the first step may be represented by a combined GDH and toxin EIA, followed by NAATs in the case of GDH-positive and toxin-negative results [28]. The IDSA and the Society for Healthcare Epidemiology of America (SHEA) recommend similar diagnostic algorithms to that of ESCMID; however, for IDSA and SHEA NAATs alone can be considered a first step method if stool samples have been previously screened from patients with suspected CDI (patients with clinical diarrhoea and history of antibiotic exposure who have not received laxatives in the past 48 h) [19,29].

Once *C. difficile* clinical disease is confirmed, a further diagnostic PCR step can be considered to detect hypervirulent strains [30]. Detection of toxigenic *C. difficile* or *C. difficile* DNA fragments is sufficient for laboratory diagnosis [31]. Diagnostics is not recommended as a test of cure in children with confirmed CDI and adequate treatment. Indeed, both toxins and *C. difficile* genetic products can still be found in patients’ stool for several weeks after resolution of symptoms, and neither EIA nor NAAT tests should be performed before 4 weeks of recovery if there is suspected recurrence [4,32].

## 3. Classifications of *C. difficile* Infection (CDI) and Treatment Strategies

We can distinguish three kinds of CDIs: (1) healthcare facility-onset (HO) CDI; (2) community-onset, healthcare facility-associated (CO-HCFA) CDI and (3) community-associated (CA) CDI. Although *C. difficile* was defined as the major cause of healthcare-associated diarrhoea in adult patients for decades [33], the rate of community-acquired CDI has increased [34]. Healthcare-associated infections remain the most frequent in adults, whereas community-associated CDI is 3-fold more common than healthcare-associated CDI in children. The increasing rate of community-acquired CDI in the paediatric population and increased rates in children with IBD and cancer have led to a shift in our understanding of the epidemiology of CDI. In children these risk factors are different than the primary risks in adults—hospitalization and prolonged antibiotic use [35].

CDI is defined as the presence of diarrhoea (3 or more loose or liquid stools in a 24-h period) or evidence of megacolon or severe ileus and either a positive laboratory diagnostic test result or evidence of pseudomembranes showed by endoscopy or histopathology (although pseudomembranous colitis is not a 100% specific marker for CDI) [19].

The first episode of CDI consists of a new primary episode of symptom onset (no episode of symptom onset with a positive result within the previous 8 weeks) and a positive assay result [17].

The first CDI should be classified, according to IDSA guidelines on CDI, by its laboratory and clinical features as A) non-severe CDI, B) severe CDI and C) fulminant CDI (Table 1) [19].

A recurrent case is defined as an episode of symptom onset and a positive assay result following an episode with a positive assay result in the previous 2–8 weeks [19]. Clinicians should guide the therapeutic strategy according to this classification.

## 4. Treatment in the Paediatric Population

Therapeutic approaches to CDI include first and second steps, which are summarized below.

### 4.1. First step: Conscientious Use of Antibiotics and Role of Probiotics

De-escalation of antibiotics that are directly associated with CDI remains a priority and should be considered as soon as possible if symptoms occur, as their use is related to a reduction in clinical response and an increase in recurrence rates. The use of penicillins, cephalosporins, clindamycin and fluoroquinolones is more commonly associated with CDI. In the paediatric population, prolonged therapy with multiple antibiotics from different classes leads to severe or fulminant infection [36].

Laboratory confirmation of CDI is needed to start empirical antibiotic therapy unless the patient presents clinical features of fulminant CDI or if it is expected to take more than 48 h to receive laboratory test results [19].

Antibiotics administration can cause antibiotic-associated diarrhoea (AAD) and for this reason de-escalation of antibiotics is extremely important. Several studies have investigated how probiotic administration could prevent AAD and CDI due to its capability of providing a gut barrier and restoring the gut microbiota [37].

In a recent review about CDI in adults, Benoit Guery et al. reported on effects of probiotics, used concurrently with antibiotics, towards preventing *C. difficile* diarrhoea [33]. A randomized, double-blinded phase 3 trial showed that *Lactobacillus* spp. administration during antibiotic therapy was associated with a lower incidence of *C. difficile*-associated diarrhoea. Two other recent probiotics were evaluated: VSL#3 and Howaru Restore—the latter was associated with a decrease in *C. difficile*-associated diarrhoea. Even if several meta-analyses indicate that probiotics may prevent CDI, there are several limitations to these meta-analyses, including differences in probiotic formulations and the duration of use, leading to insufficient data to recommend probiotic use to prevent CDI; moreover, studies are lacking in the paediatric population [33].

The effects of probiotics on AAD, but not CDI, were reviewed by Guo et al. They considered randomized, parallel, controlled trials in children (0–18 years) receiving antibiotics that compared probiotics to placebo and measured the incidence of diarrhoea secondary to antibiotic administration [38]. Evidence shows that probiotics may reduce the duration of diarrhoea by almost one day, but the benefit of high-dose probiotics (e.g., *Lactobacillus rhamnosus* or *Saccharomyces boulardii*) needs to be confirmed by large well-designed, multicentre, randomized trials. Although no serious adverse events were reported among inpatient and outpatient children in these studies, observational studies have reported serious adverse events in severely debilitated or immuno-compromised children. Therefore, the cautious use of probiotics is recommended [39].

### 4.2. Second Step: Target Specific Treatment Strategy for C. difficile Infection (CDI)

As mentioned before, the therapy is targeted according to age, severity and recurrence of the episode of infection. We can underline some differences in adult and paediatric populations, which are also due to fewer clinical trials having been conducted with children.

The first-line antibiotic therapy for the first episode of infection in the adult population is vancomycin, a glycopeptide antibiotic that inhibits bacterial wall synthesis [3]. For decades, metronidazole has been considered to be the antibiotic of choice in CDI because it was less expensive and was not associated with the potential increase in vancomycin-resistant microorganisms. Despite this, metronidazole, interacting with anaerobic host cell DNA resulting in DNA strand breakage, is rapidly absorbed and only small amounts reach the colon mucosa where CDI occurs, so its use may not be optimal for severe infections [35]. This awareness led to several studies on the use of vancomycin vs metronidazole in *C. difficile* diarrhoea. In a randomized, prospective, double-blind, placebo-controlled trial comparing vancomycin with metronidazole, patients were stratified according to disease severity [40]. The overall cure rate was 84% in the metronidazole group and 97% in the vancomycin group (*p* = 0.006). No difference was reported in patients with mild disease, but in severe forms, treatment success was seen in 76% and 97% of the cases for metronidazole and vancomycin, respectively (*p* = 0.02). This study showed that vancomycin could be superior over metronidazole for patients with severe CDI. However, recently a retrospective study of hospitalized patients with non-severe CDI showed that metronidazole was inferior to vancomycin even with respect to treatment response in this population [40]. Thus, metronidazole is not considered as a first line therapy for non-severe or severe *C. difficile* infection in the adult population.

In paediatric patients, results evaluating the optimal approach for treating an initial non-severe episode of CDI in the paediatric population are limited, and evidence of the superior effectiveness of vancomycin over metronidazole for treating paediatric CDI is not available. There are no RCTs comparing the use of these agents in children. Moreover, a recent retrospective cohort study demonstrated that vancomycin use is related to the spread of vancomycin-resistant enterococci (VRE). Metronidazole was non-inferior to vancomycin for mild CDI, but vancomycin was an independent predictor for post-CDI VRE acquisition [41]. Therefore, in the first episodes of mild acute CDI, metronidazole could be considered a valid treatment. However, according to a retrospective cohort study through which patients treated with oral vancomycin were compared to those treated with metronidazole, oral vancomycin and metronidazole impact similarly in patients’ risk of VRE. More research is needed to define clearly the impact of vancomycin and metronidazole in VRE prevalence [42].

Vancomycin is also the first choice in fulminant infections in adults [41]. It should be administered orally or by nasogastric tube. If ileus is present, consider adding rectal instillation of vancomycin. Intravenously administered metronidazole should be administered together with oral or rectal vancomycin. In the paediatric population, severe or fulminant infection should be treated in the same way. According to the American Guidelines, the first-line therapy strategy consists of oral vancomycin for 10 days with or without IV metronidazole for 10 days [17]. A recent study compared dual therapy with IV metronidazole and oral vancomycin versus vancomycin monotherapy. It assessed the prevalence of use and effectiveness of dual therapy either in non-fulminant or fulminant CDI. Dual therapy with IV metronidazole and oral vancomycin was not associated with improved outcomes compared to vancomycin alone [41]. Pending further study, IV metronidazole is still recommended for severe disease. Table 2 summarizes antimicrobial treatment recommended for CDI in the paediatric population [19]. 

### 4.3. The Role of Fidaxomicin

Fidaxomicin, discovered in the late 1970s, changed the therapeutic approach to CDI. This molecule is a macrocyclic antibiotic that inhibits bacterial RNA polymerase and has bactericidal activity. Its interesting pharmacokinetic characteristics consist of its minimal intestinal absorption and prolonged postantibiotic effect (>24 h) [42]. Fidaxomicin acts against Gram-negative anaerobes and facultative aerobes and some Gram-positive anaerobes (*C. difficile*), but it has poor activity against many other Gram-positive pathogens, such as *Staphylococcus aureus* and *Enterococcus faecium*. It forms a faecal metabolite that is active against *Clostridioides* spp. Due to its poor systemic absorption, no adjustment is needed for patients with renal or hepatic impairment. Due to its narrow antimicrobial spectrum, fidaxomicin does not affect normal intestinal flora.

Two phase 3 registration studies in adults have shown that fidaxomicin is similar to vancomycin for initial clinical cure of CDI and is associated with a significant 10–15% reduction in the risk of recurrence at 28 days after the end of treatment [35]. The efficacy of fidaxomicin on recurrence (compared with vancomycin) is due to the preservation of gut microbiota. Analysis of 89 patients from a phase 3 trial demonstrated that major components of the microbiome persisted after fidaxomicin therapy, whereas vancomycin caused a significant reduction in colony forming units of Bacteroidetes*/Prevotella* group organisms [33].

Thus, fidaxomicin is considered an equivalent alternative therapy to vancomycin in non-severe and severe treatment of CDI. This is summarized in the IDSA/SHEA 2017 guidelines, which reports that there are insufficient safety and efficacy data to recommend the use of fidaxomicin in children with CDI, although occasional off-label use has been reported [19].

Recently, the Food and Drug Administration (FDA) approved fidaxomicin for children younger than 12 years [33]. This development occurred after the SUNSHINE trial, the first prospective, multicentre, randomized, investigator-blind, phase 3 parallel-group trial that compared CDI treatment with fidaxomicin and vancomycin in a paediatric population with CDI [41]. Children with clinical criteria for CDI and who tested positive for *C. difficile* (either detection of toxins A/B or toxigenic *C. difficile* in stool) were included. Patients were randomized 2:1 to 10 days of therapy with either fidaxomicin (16 mg/kg oral suspension twice daily for patients aged 0 to <6 years, or 200 mg tablets twice daily for patients aged ≥6 to <18 years) or vancomycin (10 mg/kg oral liquid four times daily for patients aged 0 to <6 years, or 125 mg capsules four times daily for patients aged ≥6 to <18 years). No patients with IBD were included to avoid confounding from other potential diarrhoea aetiologies. The number of patients with treatment-emergent adverse events (TEAEs) appeared similar between treatment arms and between age groups [41]. While the number of patients who reached a confirmed clinical response at 2 days after the end of treatment was 77.6% (76/98) in the fidaxomicin arm and 70.5% (31/44) in the vancomycin arm, CDI recurrence before the end of the study was observed in 11.8% (9/76) of fidaxomicin-treated patients and 29.0% (9/31) of vancomycin-treated patients [43]. Therefore, as for the phase 3 fidaxomicin trial in adults, fidaxomicin was associated with a lower CDI recurrence rate in children [43]. The SUNSHINE trial will guide the design of future trials that optimize CDI case ascertainment in children and limit bias potentially related to the enrolment of those with alternative and/or concomitant diarrhoeal aetiologies in CDI [42].

The best results with fidaxomicin were obtained in children ≥2 years old and those who were diagnosed by direct toxin detection. This may be related to the high rate of asymptomatic colonization and the consequential challenge of CDI diagnosis in infants <2 years old [11].

### 4.4. Recurrences and Faecal Microbiota Transplant (FMT)

As cited before, a recurrent case is defined as an episode of symptom onset and positive assay result following an episode with a positive assay result in the previous 2–8 weeks. Although antibiotics are successful in 80–90% of initial episodes, up to 40% of children with CDI show a recurrence of infection within 60 days. Among risk factors for recurrence in children there are cancer and chronic underlying disease; in adults, recurrence is observed in old age and the use of non-CDI antibiotics during or after CDI therapy [11].

In the adult population, the first recurrence of CDI should be treated with oral vancomycin as a tapered and pulsed regimen if the first infection is treated with a 10-day course of vancomycin; otherwise, with a 10-day course of fidaxomicin if the first infection is treated with a standard 10-day course of vancomycin or a standard 10-day course of vancomycin if metronidazole is used for the primary episode [3]. Antibiotic treatment alternative options for patients with >1 recurrence of CDI include oral vancomycin with a tapered and pulsed regimen or a standard course of oral vancomycin followed by rifaximin or fidaxomicin. Faecal microbiota transplantation (FMT) is recommended for patients with multiple recurrences of CDI who have failed appropriate antibiotic treatment [3,19].

There are no well-designed trials that study the effectiveness of various treatment regimens in children with multiple recurrent CDIs. In addition, paediatric studies have not demonstrated conclusively that there is a difference in the risk of recurrence related to the therapeutic agent used to treat an initial episode. Thus, recommendations about the therapeutic approach to children with multiple recurrent CDIs must be guided by evidence shown in studies performed in adults. For children with a second recurrence of CDI who have been treated exclusively with metronidazole, oral vancomycin should be considered [3]. For children with multiple recurrences of CDI despite metronidazole and oral vancomycin, an alternate therapeutic regimen should be used [3]. FMT should be considered for children with multiple recurrences of CDI following standard antibiotic treatments [9]. Table 3 summarizes antimicrobial treatment suggested for recurrent CDI in the paediatric population [19].

Good clinical response with FMT has been observed in adults with refractory or recurrent CDI with few reports of adverse events [44,45,46]. At present, data examining the effectiveness of FMT for children are lacking. Thus, recommendations regarding the therapy in multiple recurrent CDIs in paediatric age should be guided primarily by evidence from adult studies. As recently described, FMT is the most effective therapeutic approach against recurrent CDI (rCDI) and has become a standard in therapeutic algorithms, despite its unknown long-term consequences [44,45,46].

Davidovics et al. [35] suggested FMT in children with one of the following: (1) rCDI (recurrence of symptoms within 8 weeks of treatment for CDI); (2) at least 3 episodes of mild to moderate CDI and failure of a 6- to 8-week taper with vancomycin with or without an alternative antibiotic (3) at least two episodes of severe CDI resulting in hospitalization and are associated with significant morbidity; (4) moderate CDI not responding to standard therapy for at least 1 week and (5) severe CDI or fulminant *C. difficile* colitis with no response to standard therapy after 48 h. 

Potential FMT donors should be screened and selected. Decreased microbial diversity is linked with a high risk of CDI recurrence. In CDI a microbiome with facultative anaerobes and deficient in *Bifidobacteria* and *Bacteroides* is reported. A donor microbiome with *Bifidobacteria* and *Bacteroides* would theoretically be ideal. In general, it has been suggested that adults ≥18 years old should be the ideal donors for medico-legal reasons. Donor stool should be prepared to a consistency that permits easy infusion via enema, a biopsy channel, gastrostomy tube, jejunostomy tube, nasogastric (NG), nasoduodenal or nasojejunal tube [44]. A meta-analysis including both adults and children patients did not demonstrate any significant differences in outcomes when comparing FMT for rCDI via colonoscopy versus the NG tube [45]. In a multicentre study, FMT using colonoscopy was found to be significantly more effective than FMT delivered using other routes in children [46].

FMT is safe in short-term follow-up [46], although it is not risk free. Suchitra et al. decided to conduct a study about FMT in a paediatric population and its risk of antibiotic resistance before and after the faecal transplant [35]. They applied shotgun metagenomic sequencing and advanced bioinformatic tools to the study of faecal samples from paediatric patients pre- and post-FMT and from their adult donors to describe the prevalence and potential acquisition of pathogenic microbial strains and AMR genes and to study their microbial composition and function. It was observed that, as in the adult population, FMT reduces AMR genes, and it was sustained over the follow-up period [33].

## 5. Future Perspectives

Monoclonal antibodies and vaccines represent a future perspective against CDI.

Actoxumab and bezlotoxumab, two human monoclonal antibodies targeting *C. difficile* toxins A and B, respectively, have been developed, and two phase 3 clinical trials (MODIFY I and MODIFY II) studied the two antibodies’ efficacy to decrease recurrence in 2655 patients [47,48]. In these studies, patients received standard oral antibiotics for primary or recurrent CDI with an infusion of bezlotoxumab, actoxumab plus bezlotoxumab or placebo. Bezlotoxumab alone caused a substantial reduction in recurrent infection in comparison with placebo (17% in comparison with 28%) [47]. No difference was observed in the rates of clinical cure between bezlotoxumab and placebo (80%), and sustained clinical cure was 64% for bezlotoxumab and 54% for placebo. Addition of actoxumab did not increase efficacy. A phase 3 trial in children is ongoing to analyse the safety, tolerability and efficacy of bezlotoxumab [48,49].

Regarding vaccines, studies on two different products are ongoing [3]. The first is a formalin inactivated toxoid-based vaccine obtained by inactivation of toxins A and B. A phase 1 study of the *C. difficile* toxoid vaccine was done in healthy volunteers. Vaccination was well tolerated, and more than 90% of the volunteers presented a strong serum antibody response to both toxins. Another phase 1 study studied VLA84, a recombinant fusion protein with relevant epitopes of toxins A and B, as a vaccine candidate in a healthy population and in older adults [3]. VLA84 was well tolerated and induced high antibody titres against toxins A and B in both populations.

Only further studies will permit us to understand whether these new approaches could be effective in therapy and prevention of CDI in children and adolescents.

## Figures and Tables

**Table 1:** Classification of *Clostridioides difficile* infection (CDI).

Classification	Laboratory and Clinical Features
Non-severe	Leucocytosis with a white blood cell count of ≤15,000 cells/mL and a serum creatinine level < 1.5 mg/dL
Severe	Leucocytosis with a white blood cell count of ≥15,000 cells/mL or a serum creatinine level > 1.5 mg/dL
Fulminant	Hypotension or shock, ileus, megacolon

Adapted from McDonald et al. [19].

**Table 2:** Antimicrobial treatment suggested for *Clostridoides difficile* infection (CDI) in the paediatric population.

Type of Infection	Drug	Dosage	Maximum Dose
Non-severe	Metronidazole × 10 days (PO), OR Vancomycin × 10 days (PO)	7.5 mg/kg/dose tid or qid 10 mg/kg/dose qid	500 mg tid or qid 125 mg qid
Severe/ Fulminant	Vancomycin × 10 days (PO or PR) with or without metronidazole ×10 days (IV)	10 mg/kg/dose qid 10 mg/kg/dose tid	500 mg qid 500 mg tid

Abbreviations: IV, intravenous; PO, oral; PR, rectal; qid, 4 times daily; tid, 3 times daily. Adapted from McDonald et al. [19].

**Table 3:** Antimicrobial treatment suggested for recurrent *Clostridioides difficile* infection (CDI) in the paediatric population.

Type of Recurrence	Drug	Dosage	Maximum Dose
First recurrence, non-severe	Metronidazole × 10 days (PO), OR Vancomycin × 10 days (PO)	7.5 mg/kg/dose tid or qid 10 mg/kg/dose qid	500 mg tid or qid 125 mg qid
Second or subsequent recurrence	Vancomycin in a tapered and pulsed regimen, OR Vancomycin for 10 days followed by rifaximin for 20 days, OR Faecal microbiota transplantation	10 mg/kg/dose qid 10 mg/kg/dose qid No paediatric dosing	125 mg qid 500 mg qid

Abbreviations: IV, intravenous; PO, oral; PR, rectal; qid, 4 times daily; tid, 3 times daily. Adapted from McDonald et al. [19].
